# Genetic structure of *Leptopilina boulardi *populations from different climatic zones of Iran

**DOI:** 10.1186/1472-6785-11-4

**Published:** 2011-01-27

**Authors:** Majeed Askari Seyahooei, Jacques JM van Alphen, Ken Kraaijeveld

**Affiliations:** 1 Institute of Biology, Leiden University, P.O.Box 9505, 2300 RA Leiden, the Netherlands; 2 Agricultural Research Center of Hormozgan, P.O.Box 79145/1577, Bandar Abbas, Iran; 3 Institute for Biodiversity and Ecosystem Dynamics, P.O.Box 94248, 1090 GE Amsterdam, the Netherlands

## Abstract

**Background:**

The genetic structure of populations can be influenced by geographic isolation (including physical distance) and ecology. We examined these effects in *Leptopilina boulardi*, a parasitoid of *Drosophila *of African origin and widely distributed over temperate and (sub) tropical climates.

**Results:**

We sampled 11 populations of *L. boulardi *from five climatic zones in Iran and measured genetic differentiation at nuclear (Amplified Fragment Length Polymorphism; AFLP) and mitochondrial (Cytochrome Oxidase I; COI) loci. An Analysis of Molecular Variance (AMOVA) for the AFLP data revealed that 67.45% of variation resided between populations. No significant variation was observed between climatic zones. However, a significant difference was detected between populations from the central (dry) regions and those from the wetter north, which are separated by desert. A similarly clear cut genetic differentiation between populations from the central part of Iran and those from the north was observed by UPGMA cluster analysis and Principal Coordinates Analysis (PCO). Both UPGMA and PCO further separated two populations from the very humid western Caspian Sea coast (zone 3) from other northern populations from the temperate Caspian Sea coastal plain (zone 2), which are connected by forest. One population (Nour) was genetically intermediate between these two zones, indicating some gene flow between these two groups of populations. In all analyses a mountain population, Sorkhabad was found to be genetically identical to those from the nearby coastal plain (zone 2), which indicates high gene flow between these populations over a short geographical distance. One population from the Caspian coast (Astaneh) was genetically highly diverged from all other populations. A partial Mantel test showed a highly significant positive correlation between genetic and geographic distances, as well as separation by the deserts of central Iran. The COI sequences were highly conserved among all populations.

**Conclusion:**

The Iranian populations of *L. boulardi *showed clear genetic structure in AFLP profiles, but not in COI sequence data. The transfer of fruits containing *Drosophila *larvae parasitized by *L. boulardi *appears to have caused some unexpected gene flow and changed the genetic composition of populations, particularly in urban areas. Nevertheless, our results suggest that climate, geographic distance and physical barriers may all have contributed to the formation of genetically distinct populations of *L. boulardi*. Inevitably, there will be overlap between the portions of variance explained by these variables. Disentangling the relative contributions of climate and geography to the genetic structure of this species will require additional sampling.

## Background

Changing climates are expected to have profound effects on the genetics of insect populations. One way to study such effects is to compare populations of a widely distributed organism across contrasting climates.

One of the main constraints on local genetic differentiation and adaptation is extensive gene flow between populations. Reduced dispersal between populations can lead to genetic subdivision of populations [1] and may facilitate local adaptation. Environmental or physical barriers may promote isolation of populations. These include geographic distance [2] and physical barriers like mountains, rivers and stretches of unsuitable habitat.

DNA markers provide powerful and efficient tools to study genetic diversity in insect populations [3-5] at both inter- and intraspecific level. Amplified Fragment Length Polymorphism (AFLP) is a useful DNA fingerprinting technique to study genetic diversity within a species [6-9] because it allows detection of genetic variation of organisms based on DNA from any source and complexity [10] without prior knowledge of the gene structure or sequences. AFLP markers have been used to infer the role of geographical distance and barriers to gene flow in shaping the genetic structure of population [e.g., [11-13]] in a variety of organisms. The results of AFLP analysis can be translated to the genetic distances [4,8,14] of populations. Genetic distance of isolated population is the most fundamental information need to assess the role of geographical barriers and distances in genetic diversification of populations. Mitochondrial markers have also been used in population genetic studies [e.g., [15-17]] of insects. For example, in the ant *Leptothorax rugatulus *[16] a mitochondrial marker was found to be more informative than microsatellites and discriminated significantly better between populations. Using mitochondrial markers may provide valuable information on the migration of females between populations because mitochondria are maternally inherited.

**Figure 1:**
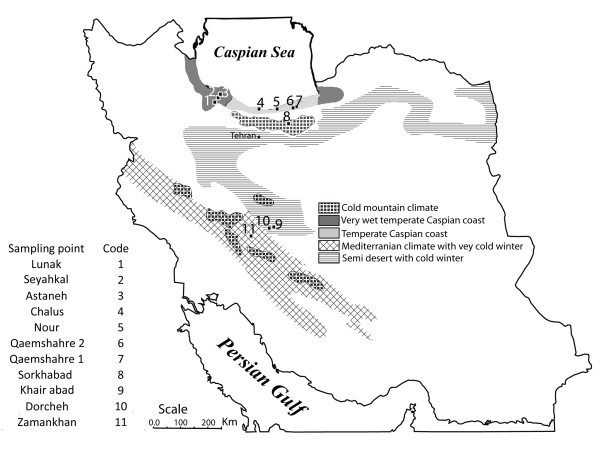
**Map of the Islamic republic of Iran with climate zones indicated in shades of grey and patterns**. Sampling points are indicated by the code name of populations provided in the legend.

Insect parasitoids have been a favorite model in ecology and evolutionary biology [18] studies. *Drosophila *parasitoids have played a major role in these studies, because of their ease of maintenance in the laboratory, the enormous biological and genetic information on their *Drosophila *host, which has been used as model organism for almost a century [19] and because of their diversity which allows comparative studies. In the present study we used AFLP markers and mitochondrial gene sequencing to investigate the role of geographical distance in shaping genetic variation in Iranian *Leptopilina boulardi *(Hymenoptera: Figitidae) populations. *L. boulardi *is a larval parasitoid of *Drosophila *of African origin [20] which is widely distributed over tropical and warm temperate regions. We used both AFLP and COI gene sequencing to study the genetic structure among 11 populations of *L. boulardi *collected from five contrasting climate zones in Iran (Figure 1, Table 1). The zones were chosen to represent five distinct climates varying in precipitation, length of season and minimum and maximum temperatures. The main goal of this study was to investigate to what extent the genetic structure of these populations matched climatic and geographical patterns.

**Table 1:** Description of the trapping locations of the populations including climate, vegetation and elevation

Zone	Population ID	Climate	Description of the location	Vegetation	Elevation (m)
1	Sorkhabad	Mountain	In the northern slope of the Damavand mountain, toward Mazandaran province	Tree and bushes along the river	1975

2	Qaemshahr 1	Temperate wet forest	In Mazandaran province, coastal Caspian sea	Forest	76

2	Qaemshahr 2	Temperate wet forest	In Mazandaran province in coastal Caspian sea	Forest	75

2	Nour	Temperate wet forest	In Mazandaran province in coastal Caspian sea	Natural park	20

2	Chalus	Temperate wet forest	In Mazandaran province in coastal Caspian sea	Forest	76

3	Astaneh	Temperate very wet forest	In Gilan province in coastal Caspian sea	Windbreak of rice field, close to city	8.5

3	Seyahkal	Temperate very wet forest	In Gilan province in coastal Caspian sea	Very dense forest	378

3	Lunak	Temperate very wet forest	In Gilan province in coastal Caspian sea	Very dense forest	485

4	Dorcheh	Semi dry desert with cold winter	Close to Esfahan along the Zayandehrod river	Orchard along the river	1625

4	Khairabad	Semi dry desert with cold winter	Close to Esfahan along the Zayandehrod river	Orchard and field along the river	1605

5	Zamankhan	Mediterranean with very cold winter	Close to Shahre kord and along Zayandehrod river	Orchard and field along the river	1873

## Results

### AFLP analysis

**Figure 2:**
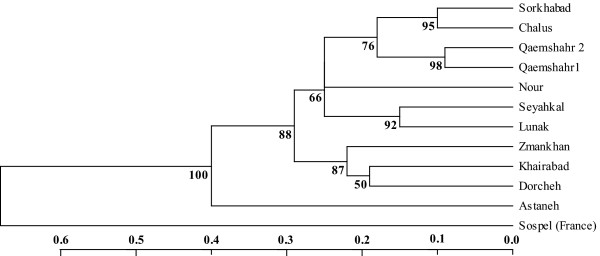
**Dendogram derived from the UPGMA (unweighted pair group methods of arithmetic averages) analysis of 147 polymorphic AFLP bands**. Shown are the genetic distances of 11 population of Iranian *Leptopilina boulardi *and their outgroup (a population of *L. boulardi *from France). Scales indicate genetic distances (Nei& Li, 1979) and the numbers at nodes represent bootstrap value (1000 replicates).

The 9 primer combinations yielded 670 scorable bands of which 147 (21.94%) were polymorphic. AMOVA of the AFLP results revealed that the main proportion of genetic variation resided among populations (67.45%, P < 0.001). Pair-wise differences of *F*_*st *_values among populations ranged from 0.53 - 0.94 (Additional file 1). No significant differences were observed between populations from different climate zones. However, a significant proportion of variation was explained by the distinction between central and northern populations (18.1%, P = 0.017). Consistent with AMOVA, UPGMA tree and bootstrap analysis indicated that the three populations from the central part of Iran (Dorcheh, Khairabad and Zamankhan) clustered together, but were differentiated from the northern populations sampled along the Caspian Sea coast (bootstrap value = 87%, Figure 2). One population from the very wet Caspian Sea coast (Astaneh) was considerably different from all other populations. In pair-wise comparison of population the highest *F*_*st *_values was observed when we compared this population with the others. UPGMA tree also separated this population strongly from all other with high bootstrap value (bootstrap = 88). The Sorkhabad population, collected from a mountainous region on the slope of the Damavand mountain near Mazandaran clustered together with the wet Caspian Sea coastal populations. Consistent with UPGMA tree and PCO plots, low *F*_*st *_values in pair-wise comparison were observed when we compared Sorkhabad with other northern populations (e.g. Chalus: *F*_*st *_= 0.53).

**Figure 3:**
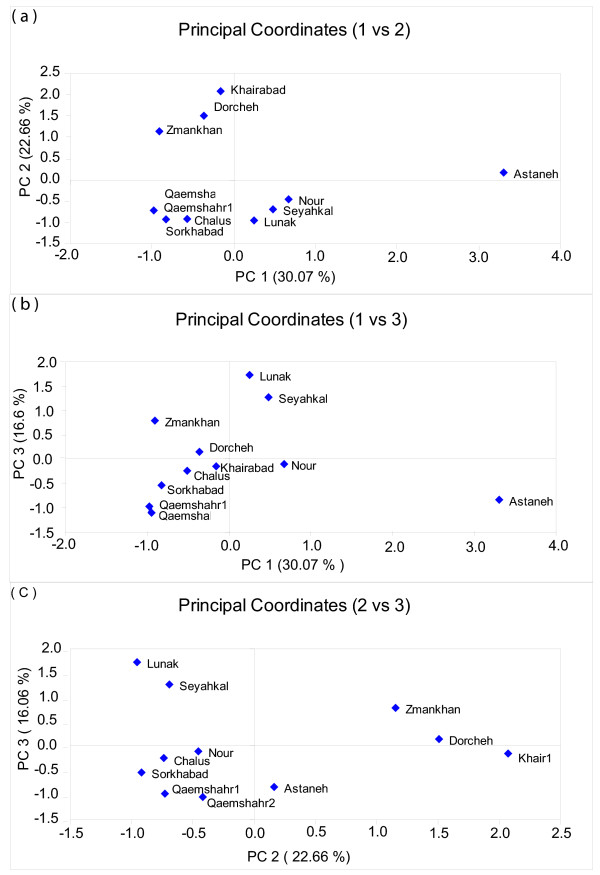
**Separation of Iranian *L. boulardi *populations by the first three coordinates in Principal Coordinates analysis**. The first three coordinates explained 69.23% of AFLP variations among Iranian *L. boulardi *populations. For the origin zone of populations see table 1.

Principal Coordinates Analysis (PCO) revealed informative separation of populations (Figure 3). The first three coordinates explained 69.32% of AFLP variation. By plotting the first two coordinates (which together explained 52.73% of the AFLP variation) the three populations from the dry central region of Iran clustered separately from the northern populations. These two principal coordinates also separated the populations from the two northern zones (zone 2 and 3), except Nour - a population from zone 2 clustered as zone 3 and Astaneh - the most divergent population which stood apart from all others. The other two PCO plots showed further evidence for divergence among the populations along the Caspian Sea coast. By plotting the first and third coordinates and second and third coordinates (which in total explained 46.67% and 39.26% of AFLP variation, respectively) two clusters were evident among the populations from the Caspian Sea coast, while Astaneh, the most divergent population, again stood apart from all other groups. Furthermore, the Zamankhan population from a Mediterranean climate (zone 5) appeared separated from the two other central populations from zone 4 (Khairabad and Dorcheh). Consistent with the UPGMA analysis, the Sorkhabad population from the mountain region clustered with populations from the geographically close, but ecologically different wet Caspian Sea coast.

### Isolation of populations by distance

**Figure 4:**
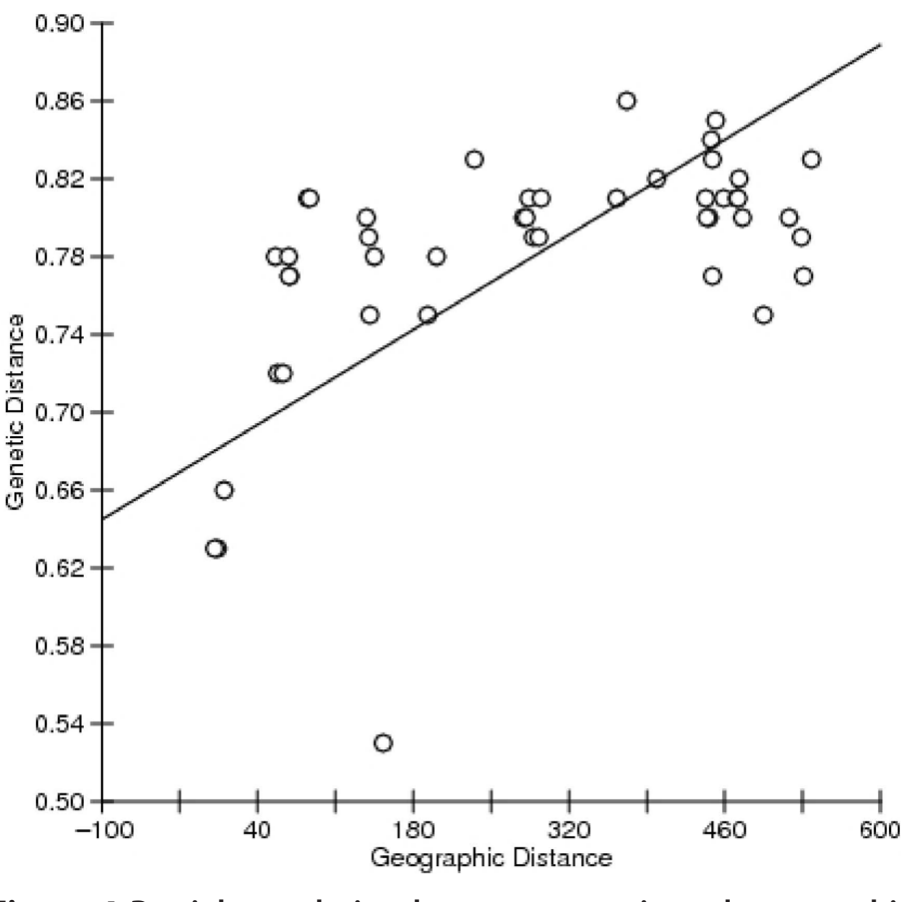
**Partial correlation between genetic and geographic distances, excluding Astaneh, the most divergent population in all analyses**.

A significant positive correlation between genetic and geographic distance was observed among the *L. boulardi *populations (Mantel test; r = 0.47, P < 0.001). The result of this test showed that a considerable part of the genetic variation was explained by geographic distance and supported the UPGMA and Principal Coordinate analyses since all geographically-close populations resembled each other. The only exception was the population from Astaneh, which was highly distinct from all others populations, even from those collected from a distance of less than 15 km. A partial Mantel test including all populations found no significant effect for isolation by the deserts of central Iran (mantel test; r = 0.12, P = 0.25). However, by excluding the Astaneh population from the partial Mantel test, we found a significant effect for isolation by dry desert (mantel test; r = 0.38, P = 0.001). After correcting for the effect of isolation by desert, the partial correlation between geographic and genetic distances decreased somewhat, but still remained highly significant (mantel test; r = 0.41, P = 0.011, Figure 4).

### Variation in cytochrome oxidase I (COI)

COI sequence was found to be highly conserved among the Iranian *L. boulardi *populations. Only two bases were found to be different in two Caspian Sea coast populations and four insertions and one replacement were found in the out-group compared to the Iranian populations (Table 2).

**Table 2:** Polymorphic site of the COI gene in 638 bp sequenced for 11 Iranian *Leptopilina boulardi *aligned with their out-group, Sospel- a population of *L. boulardi *from France.

	Position						
**Population**	**10**	**60**	**131**	**321**	**338**	**440**	**459**

Sospel	-	G	C	G	A	A	C

Zamankhan	-	-	C	-	-	G	-

Qaemshahr1	-	-	C	-	-	G	-

Qaemshahr2	-	-	C	-	-	G	-

Khairabad	-	-	C	-	-	G	-

Dorche	-	-	A	-	-	G	-

Nour	-	-	C	-	-	G	-

Astaneh	-	-	C	-	-	G	-

Seyahkal	-	-	C	-	-	G	-

Lunak	-	-	C	-	-	G	-

Sorkhabad	-	-	C	-	-	G	-

Chalus	C	-	C	-	-	G	-

## Discussion

The AFLP analysis provided enough information to allow a clear distinction among *L. boulardi *populations originating from different climatic zones in Iran. With the exception of one population (Astaneh), the genetic distances between populations correlated closely with geographic distances. A partial Mantel test revealed a significant correlation between habitat isolation and genetic diversification. AMOVA also indicated diversification of populations originating from distinct dry or wet regions. Populations collected within short proximity tended to resemble each other genetically (e.g. Qaemshahr1 and Qaemshahr 2; distance 1.5 km), as expected because of the high likelihood of gene flow. This was true even for populations that were ecologically very different: the mountain population of Sorkhabad was genetically indistinguishable from populations from the nearby coastal plain. The partial Mantel test showed significant effects of geographical barriers as well as the distance on genetic divergence. The positive correlation between genetic and geographic distances in our study is consistent with several other genetic structure studies [11,13,21] on insect populations. However, studies on the genetic structure of *Drosophila *parasitoid populations are rare. A comparison of *L. clavipes *populations in western Europe found distinct genetic differentiation between sexual and asexual populations [22], but no correlation between geographic and genetic distances.

The different climatic environments we sampled (including desert, Mediterranean, and wet temperate conditions) undoubtedly impose different selection pressures on the life history of parasitoids. The genetic differences we observed between geographically isolated populations imply low gene flow. This will facilitate genetic response to such selection.

In addition to geography and climate, human activity also appears to affect the genetic structure of *L. boulardi *populations. Parasitized fruit fly larvae may be transported with fruits and give rise to populations dissimilar from the neighbouring ones, in particular in urban areas. We found unexpected genetic divergence in two northern populations Astaneh (which was highly divergent from the neighbouring) and Nour (which showed intermediate structure between two Caspian Coast zones). Both populations originated from urban areas and could have arrived with fruit transports.

In addition to geography and climate, variation in hosts may result in adaptation and genetic differentiation of parasitoids [23], due to the intimate relationship between host and parasitoid. However, we found no evidence of the differentiation caused by hosts in this study.

Populations in the central part of Iran were sampled from isolated locations surrounded by dry desert. The relatively high genetic divergence over relatively short geographic distance between these central populations compared to northern populations implies that the barrier formed by unsuitable habitat may contribute to genetic diversification independently of geographic distance (Figure 1, 2; compare geographic and genetic distances between Zamankhan and Dorcheh or Khairabad in central Iran to those between Qaemshahr1,2 and Chalus in the north).

Sequence data on the cytochrome oxidase I gene were less informative in our study than ALFPs. Mitochondrial markers are potentially informative markers in genetic studies of insect populations [15-17], sensitive to differences in the rates of male and female migration [24] between populations. However, most studies using CO I and II as molecular markers in insects have used them in phylogenetic and taxonomic contexts to discriminate between higher taxa [24-26] than populations. Our results demonstrate a highly conserved pattern for COI among *L. boulardi *populations.

## Conclusion

In summary, this study provides strong evidence for genetic divergence of geographically isolated populations of *L. boulardi *from different climate zone of Iran. Geographic distance by itself explained a large amount of the variance in AFLP profiles. The two most distinct clusters, namely populations from central Iran versus those from the north, are also separated by a large area of unsuitable habitat, suggesting that such barriers may further contribute to genetic divergence. The mountain population from Sorkhabad showed that differences in ecology by themselves are not enough to overcome the homogenising effect of gene flow over short geographic distances. Climate may drive host distribution patterns and select for differences in life-history traits of parasitoids. The reduction in gene flow between geographically isolated populations will facilitate genetic adaptation to such selection pressures.

## Methods

### Field sampling

*Leptopilina boulardi *populations were sampled along a climatic cline stretching from the northern to the central regions of the Islamic Republic of Iran. The transect covered five climatic zones as follows (Table 1, Figure 1): cool mountains in Damavand (zone 1), wet forests (zone 2) and very wet rain forests along the Caspian sea coast (zone 3), a dry and hot climate with cold winters in the Esfahan region (zone 4) and a Mediterranean climate with very cold winters in Shahre Kord (zone 5). Sampling was conducted in mid-summer (July 2006) to increase the likelihood of collecting adult wasps from all the zones. At each sampling site, we placed 12 traps. The traps consisted of plastic containers (diameter 10 cm; height 7.5 cm) with a 3 cm diameter circular hole in the lid, which was covered with a plastic mesh wide enough for *Drosophila *flies and their parasitoids to enter the traps. Several layers of filter paper were placed in each trap to absorb water and provide pupation sites for *Drosophila *larvae. The traps were baited with a piece of banana and suspended from trees and its position was recorded by GPS (Additional file 2). After a week the traps were collected and all pupae in the traps were wrapped in filter paper and transferred to the lab. *Leptopilina boulardi *was the only parasitoid collected from all locations. Partially inbred lines were set up from each sampling site and 20 female wasps per population from early generations were frozen at -80°C for genetic analysis.

### DNA extraction

Five female wasps in four replications were pooled per strain for DNA extraction using an adapted CTAB protocol for insects [27], including an extra RNase A step. The quality of the extracted DNA was checked on a 0.8% agarose gel and its quantity measured by a spectrophotometer (ND-1000, http://www.nanodrop.com ). In the case of low quantity or sheared DNA the DNA extraction was repeated using five new females from the same strain.

### AFLP analysis

To assess the genetic diversity of the *L. boulardi *populations, we employed the Amplified Fragment Length Polymorphism (AFLP) technique [10] with a slight modification to the standard procedure. Approximately 500 ng of genomic DNA was incubated with EcoRI and MseI enzymes (New England Biolabs:http://www.neb.com). EcoRI/MseI adaptors were ligated to the restriction fragments. Preamplification was conducted using EcoRI+A and Mse+C primers (Table 3). A touchdown profile amplification during which the annealing temperature was dropped by 0.7°C in each cycle was used to increase the optimal primer selectivity in this step. Selective amplification was performed using one of three EcoRI primers (fluorescently labeled) and one of three MseI with three and two base extensions in each primer respectively. Different combinations of these primers resulted in 9 useful primer combinations (Table 4).

**Table 3:** List of primers and adaptor used for the AFLP analysis

Type	Name	Sequence 5'-3'
Adaptor	EcoRI adaptor/F	CTCGTAGACTGCGTACC
	EcoRI adaptor/R	AATTGGTACGCAGTCTAC
	MseI adaptor/F	GACGATGAGTCCTGAG
	MseI adaptor/R	TACTCAGGACTCAT

Primer, pre select. amp.	EcoRI Preamp.	GAC TGCGTACCAATTCA*
	MseI Preamp.	GATGAGTCCTGAGTAAC*

Primer, select. amp	EcoRI Select.	GACTGCGTACCAATTC**A**NN *
.	MseI Select.	GATGAGTCCTGAGTAA**C**N *

*Nucleotides in bold are the fixed extended base in the 3' end of the primers and N are the variable extension to provide different primer combination, see above.

**Table 4:** List of selective amplification primers and fluorescent labels used in nine different combinations and the number of scorable and polymorphic bands generated by each combination.

Combination	Primers and labels	Scored band	polymorphic band
A	EcoRI+ACA (labeled Fam), MseI+CA	117	22 (18.8%)
B	EcoRI+AGG (labeled Joe), MseI+CA	63	20 (31.74%)
C	EcoRI+AAC (labeled Ned), MseI+CA	65	12 (18346%)
D	EcoRI+ACA (labeled Fam), MseI+CT	101	15 (14.85%)
E	EcoRI+AGG (labeled Joe), MseI+CT	65	25 (38.46%)
F	EcoRI+AAC (labeled Ned), MseI+CT	58	11 (18.96)
G	EcoRI+ACA (labeled Fam), MseI+CG	80	14 (17.5%)
H	EcoRI+AGG (labeled Joe), MseI+CG	69	16 (23.19%)
I	EcoRI+AGG (labeled Joe), MseI+CC	52	12 (23.08%)
Total band		670	147

PCR products from the selective amplification were purified run on a DNA sequencher MegaBACE™ 1000 system (Amersham Pharmacia USA, http://www.gelifesciences.com/aptrix/upp01077.nsf/Content/life-sciences_homepage at 10 kV for 75 min. The fluorescent profiles were loaded into Fragment Profiler™ software ver1.2 (Amersham, Biosciences, http://www.amersham.com) using specific peak filters and were manually checked for correct alignment of the size standard. The positions of polymorphic markers between 80 and 500 bp were scored and exported as a binary matrix in an excel sheet.

### Amplification and sequence alignment for COI

A 638 bp portion of the cytochrome oxidase subunit I (COI) gene was amplified for three individuals per strain to evaluate the mitochondrial variation among the 11 *Leptopilina boulardi *populations from Iran. A *L. boulardi *population from Sospel (France) was used as out-group. PCR amplification was performed using a forward primer designed by Scheffer and Grissell [28], COI-1775-F 5^'^-CGAATAAATAATATAAGATTTTG-3^' ^and a reverse primer designed for *Leptopilina clavipes *(K. Kraaijeveld, unpublished), COI-2413-R, 5^'^-TCATCTAAAAATTTTAATCCCAGT-3^'^. The amplification was carried out on a Thermocycler PTC-2000 using the following thermal cycles: 3 min at 92°C followed by a touchdown with one degree drop in annealing temperature per cycle from 53-40°C (10 sec at 92°C, 10 sec at 53-40°C and 2 min at 72°C), then 25 cycles of 10 sec at 92°C,10 sec at 40°C and 2 min at 72°C, ending with 5 min extension at 72°C. Each PCR reaction contained 1.2 μl of 2.5 mM DNTPs, 0.5 μl of 10 μM of each primer, 0.15 μl of Taq DNA polymerase(5 U), 1.5 μl of 10X Buffer and 0.45 μl of 15 mM MgCl2 μl; all Qiagen products and was adjusted to 15 μl with autoclaved nanopure water. The PCR products were purified using Wizard SV Gel and PCR Clean-Up System (Promega, http://www.promega.com ) following the manufacturer's protocol. The amplified bands were sequenced both forward and reverse on MegaBACE™ 1000 sequencher. The Sequencher software Version 4.2 (Gene Codes Corp.) was used to assemble the contigs and obtain consensus sequences. The sequences were aligned using pairwise-alignment in MacClade 4.08 [29] and edited manually.

### Data analysis

Population structure was investigated using a molecular variance software package (AMOVA) and *F*_*st *_statistics were estimated using ARLEQUIN software [30,31], ver. 3.5. Pair-wise genetic distances between populations were calculated from the AFLP data using Nei and Li's index [32] in Treecon version 1.3b [33] for windows. UPGMA cluster analysis [34] with 1000 replications of bootstraps was performed in Treecon and genetic distances were visualized in a dendrogram format. The geographic distances between collecting sites were calculated from their GPS locations and combined with the genetic distance values into a pair-wise genetic and geographic distance matrix. We set an indicator as isolated (1) or connected (0) for the isolation by unsuitable habitat in to 0/1 binary character matrix. Isolation by distance and geographical barrier was investigated with a partial Mantel test [35] using the web-based program Isolation by distance web service (IBDWS) [36] version 3.16. The combined binary AFLP data matrix was used to perform Principal Coordinate analysis (PCO) with GenAlex 6 [37] using Nei and Li's coefficient [32] for calculating similarities, three first coordinates were used to graphically depict genetic variation among populations.

## Authors' contributions

MAS designed the project, collected samples in the field collected and analysed the data and wrote the manuscript. JJMvA participated in the field work, supervised project and improved the manuscript. KK helped in improving methodology and data analysis, supervised the project and improved the manuscript. All authors read and approved the final version.

## Supplementary Material

Additional file 1 **Fst and P value for pair wise comparison of populations**. Below diagonal Fst value and above P value of pair wise comparison of populations derived from AMOVA of AFLP results of eleven populations of *Leptopilina boulardi *from Iran. Click here for file

Additional file 2 **Sampling points**. Sampling points and their GPS coordinates, climate zone and elevation above sea level. Click here for file
