# Supplementary material for: Genetic structure of Leptopilina boulardi populations from different climatic zones of Iran
Source: BMC Ecol. 2011 Jan 27;11:4. doi: 10.1186/1472-6785-11-4 (PMC3042369; doi:10.1186/1472-6785-11-4)
Supplement: Additional file 1 — Fst and P value for pair wise comparison of populations. Below diagonal Fst value and above P value of pair wise comparison of populations derived from AMOVA of AFLP results of eleven populations of Leptopilina boulardi from Iran. [file 1472-6785-11-4-S1.DOC]

Additional file 1

Title: Fst and P value for pair wise comparison of populations

Description: Below diagonal Fst value and above P value of pair wise comparison of populations derived from AMOVA of AFLP results of eleven populations of *Leptopilina boulardi* from Iran.

| Pop. | Lunak | Seyahkal | Astaneh | Chalus | Nour | Qaemshar1 | Qaemshar2 | Sorkhabad | Khairabad | Dorcheh | Zamankhan |
| --- | --- | --- | --- | --- | --- | --- | --- | --- | --- | --- | --- |
| Lunak | NA | 0.04 * | 0.027* | 0.027* | 0.018* | 0.04 * | 0.028* | 0.027* | 0.027* | 0.036* | 0.027* |
| Seyahkal | 0.63 | NA | 0.04 * | 0.018* | 0.027* | 0.045* | 0.054 N.S | 0.018* | 0.026* | 0.038* | 0.026* |
| Astaneh | 0.91 | 0.92 | NA | 0.036* | 0.009** | 0.046* | 0.009** | 0.036* | 0.009** | 0.09N.S | 0.036* |
| Chalus | 0.75 | 0.78 | 0.94 | NA | 0.027* | 0.047* | 0.046* | 0.056 N.S | 0.024* | 0.027* | 0.035* |
| Nour | 0.75 | 0.78 | 0.92 | 0.82 | NA | 0.018* | 0.07 N.S | 0.018* | 0.008** | 0.029* | 0.054 N.S |
| Qaemshar1 | 0.80 | 0.79 | 0.93 | 0.80 | 0.81 | NA | 0.047* | 0.046* | 0.009** | 0.036* | 0.024* |
| Qaemshar2 | 0.80 | 0.81 | 0.94 | 0.79 | 0.81 | 0.63 | NA | 0.037* | 0.04 * | 0.016* | 0.025* |
| Sorkhabad | 0.79 | 0.63 | 0.94 | 0.53 | 0.78 | 0.80 | 0.77 | NA | <0.001*** | 0.018* | 0.036* |
| Khairabad | 0.79 | 0.78 | 0.90 | 0.79 | 0.81 | 0.79 | 0.80 | 0.81 | NA | 0.035* | 0.032* |
| Dorcheh | 0.83 | 0.83 | 0.94 | 0.86 | 0.83 | 0.83 | 0.84 | 0.86 | 0.66 | NA | <0.001** |
| Zamankhan | 0.82 | 0.80 | 0.93 | 0.82 | 0.81 | 0.81 | 0.80 | 0.82 | 0.72 | 0.72 | NA |
