# Supplementary material for: Genetic structure of Leptopilina boulardi populations from different climatic zones of Iran
Source: BMC Ecol. 2011 Jan 27;11:4. doi: 10.1186/1472-6785-11-4 (PMC3042369; doi:10.1186/1472-6785-11-4)
Supplement: Additional file 2 — Sampling points. Sampling points and their GPS coordinates, climate zone and elevation above sea level. [file 1472-6785-11-4-S2.DOC]

Additional file 2

Title: Sampling points.

Description: Sampling points and their GPS coordinates, climate zone and elevation above sea level.

| Location | Altitude | Latitude | Elevation (m) | Zone |
| --- | --- | --- | --- | --- |
| Lunak | N: 37, 00.656 | E: 49, 51.838 | 486 | 3 |
| Seyahkal | N: 37, 01.561 | E: 49, 52.845 | 379 | 3 |
| Astaneh | N: 37, 15.592 | E: 49, 36.188 | 9 | 3 |
| Chalus | N: 36, 38.131 | E: 51, 24.698 | 77 | 2 |
| Nour | N: 36, 34.922 | E: 52, 02.839 | 20 | 2 |
| Qaemshahre 1 | N: 36, 27.744 | E: 52, 56.161 | 94 | 2 |
| Qaemshahre 2 | N: 36, 27.613 | E: 52, 56.693 | 118 | 2 |
| Sorkhabad | N: 35, 44.968 | E: 52, 45.998 | 1935 | 1 |
| Khairabad | N: 32, 31.345 | E: 51, 30.567 | 1627 | 4 |
| Dorcheh | N: 32, 35.396 | E: 51, 33.042 | 1605 | 4 |
| Zamankhan | N: 32, 29.792 | E: 50, 53.933 | 1874 | 5 |
